# Influence of Experimental Warming on the Rate and Duration of Fruit Growth and Oil Accumulation in Young Olive Trees (cvs. Arbequina, Coratina)

**DOI:** 10.3390/plants12101942

**Published:** 2023-05-10

**Authors:** Andrea Miserere, Peter S. Searles, M. Cecilia Rousseaux

**Affiliations:** 1 Centro Regional de Investigaciones Científicas y Transferencia Tecnológica de La Rioja (CRILAR-Provincia de La Rioja-UNLaR-SEGEMAR-UNCa-CONICET), Entre Ríos y Mendoza s/n, Anillaco 5301, La Rioja, Argentina; amiserere@conicet.gov.ar (A.M.); psearles@conicet.gov.ar (P.S.S.); 2 Instituto de Investigación y Desarrollo Agropecuario (IIDA), Departamento de Ciencias y Tecnologías Aplicadas (DACTAPAyU), Universidad Nacional de La Rioja (UNLaR), Av. Luis M. de la Fuente s/n, Ciudad Universitaria de la Ciencia y de la Técnica, La Rioja 5300, La Rioja, Argentina; 3 Departamento de Ciencias Exactas, Físicas y Naturales (DACEFyN), Universidad Nacional de La Rioja (UNLaR), Av. Luis M. de la Fuente s/n, Ciudad Universitaria de la Ciencia y de la Técnica, La Rioja 5300, La Rioja, Argentina

**Keywords:** fatty acids, fruit dry weight, global warming, oil concentration, *Olea europaea*, open top chambers

## Abstract

Olive tree cultivation in new warmer areas and climate change have increased the global interest in understanding how air temperature affects both fruit growth and oil accumulation. The aims of this study were to evaluate the rate and duration of fruit growth and oil accumulation in response to experimental warming (+3) in a semiarid region of Argentina; and assess how warming affected fatty acid composition. Young, potted olive trees (cvs. Arbequina, Coratina) were warmed (T+) or maintained near ambient temperature (T0) inside open top chambers in the field during oil accumulation in 2014–2015 or 2015–2016 using different trees in each season. Warming reduced the rate of both fruit growth and oil accumulation in T+ compared to T0 in both cultivars. These rate reductions under T+ led to large decreases in final fruit dry weight and oil concentration. In contrast, the durations (i.e., days) of fruit growth and oil accumulation were most often not affected. Cultivar x temperature interactions were observed in 2014–2015 with warming decreasing oleic acid and increasing linoleic acid in cv. Arbequina, while cv. Coratina showed no response to warming. However, no interactions were found in 2015–2016. Studying how fruit growth and oil accumulation respond to adaptation strategies against increasing air temperatures should be a priority in both young and mature olive trees of numerous cultivars given crop expansion to new regions and future climate scenarios.

## 1. Introduction

Olive (*Olea europaea*) is one of the main oil crops worldwide due to its high oil quality for human consumption. Olive trees are well adapted to the prevailing environmental conditions in the Mediterranean Basin, where this iconic fruit tree was domesticated more than 5000 years ago [1]. In the last few decades, olive cultivation has expanded to warmer areas, and climate change has increased the overall global interest in understanding how environmental factors affect olive trees and their yields [2,3]. In particular, high air temperature during fruit growth and ripening has been observed to reduce oil yield and quality including changes in fatty acid profile (e.g., [4,5,6]), although the mechanisms behind these responses are still not clear [7]. In this context, a better understanding of how air temperature affects fruit growth and oil accumulation is crucial for maintaining crop production. In addition, establishing strategies to reduce canopy temperatures that allow adaptation of olive production to warmer climate scenarios will likely be necessary [8,9,10].

In olive, multi-site and/or multi-year comparisons have been widely used and have provided some insight into the responses of genotype, environment, and their potential interactions on fruit growth and oil yield and quality [11,12,13,14,15,16]. Such comparisons suggest that differences in olive fruit weight due to environment are often a function of temperature [4], and that the differences are more strongly related to fruit growth rate (i.e., fruit growth per day) than to the growth period duration (i.e., number of days) [11]. In terms of oil accumulation, maximum oil concentration was highly related to oil accumulation rate in olive fruit in Argentina [4], although the duration of oil accumulation has also been shown to be of significance [11]. A recent study in southern Spain observed that maximum oil% for a given genotype depended on the specific environmental conditions (i.e., a genotype x environment interaction, GxE), while the oil accumulation rate and the date of maximum oil% appeared to depend predominantly on the environment [13]. Another recent study in Israel used an interesting approach of growing young potted trees of several cultivars at both a moderate and a warm temperature site to assess oil accumulation [15]. Despite the important contribution of such multi-site studies in better understanding fruit growth and oil accumulation, the effects of temperature and other environmental (i.e., solar radiation, soil type) factors cannot be fully separated. Differences in orchard design and crop management between orchards can also not be easily taken into account in multi-site studies. Thus, more direct methods based on manipulative warming experiments that ensure more controlled growth conditions would likely provide valuable information for improving our knowledge. 

The number of studies that have actively warmed plant organs or whole olive trees in the field is still limited. Fruit-bearing branches have been actively warmed within small, transparent chambers using electrical resistors during the fruit growth period. In one study, it was found that oil concentration decreased when mean daily air temperature increased from 16 to 32 °C, while fruit dry weight only decreased above 25 °C [5]. To more closely imitate global warming, whole olive trees have also been actively warmed by 4 °C above the outdoor temperature during the entire growing season using open top chambers (OTCs) in southern Spain [17,18]. Elevated temperature appeared to strongly reduce fruit set and number as well as fruit weight and oil concentration, although control chambers with otherwise similar microclimatic conditions (i.e., wind, solar radiation) were not employed. 

Using both control and actively warmed OTCs (3–4 °C above control) in northwest Argentina, several physiological and production-related variables were evaluated during the oil accumulation phase after final fruit set [6,19,20]. The maximum leaf photosynthetic rate was not significantly affected when warming was applied during that phase for a single season due to thermal acclimation, although large increases in leaf and whole plant transpiration were reported [21,22]. Despite no apparent decrease in photosynthesis, reductions in final fruit dry weight and oil% were prominent, which led to decreases in oil yield per tree in both cvs. Arbequina and Coratina [6]. These reductions coincided with a shift towards more vegetative growth in the warmed OTCs. 

The fatty acid (FA) profile is one of the principal components of olive oil quality. Oleic acid is the main fatty acid (55–83% of total FA content) and high oleic acid is associated with better oil nutritional value and shelf life. Although genotype mostly determines the FA profile, it also depends on fruit ontogeny and environment with management practices having a limited role [12,23,24,25]. In warm regions or years, olive fruit present a lower oleic acid content and higher palmitic and linoleic acid contents in many regions [4,26,27,28]. Furthermore, the environmental response of fatty acid composition can include GxE interactions [4,15,25]. To the best of our knowledge, no previous studies have considered the direct effects of warming on FAs (%) in controlled field experiments. 

Given the overall importance of understanding the role of air temperature on fruit and oil production in olive trees, the aims of the current study were to: (1) evaluate the rate and duration of fruit growth and oil accumulation in response to experimental warming using open top chambers in a semiarid, warm region of Argentina; and (2) assess how warming affected fatty acid composition. To facilitate these aims, warming was applied during the entire oil accumulation phase in young olive trees of two olive cultivars (cvs. Arbequina, Coratina), and fruit were sampled at regular intervals during this phase. The cultivars were chosen for their contrasting fruit weight and reported differences in fatty acid profile stability in response to environment [4,29].

## 2. Results

### 2.1. Air Temperature and Other Environmental Variables 

**Figure 1:**
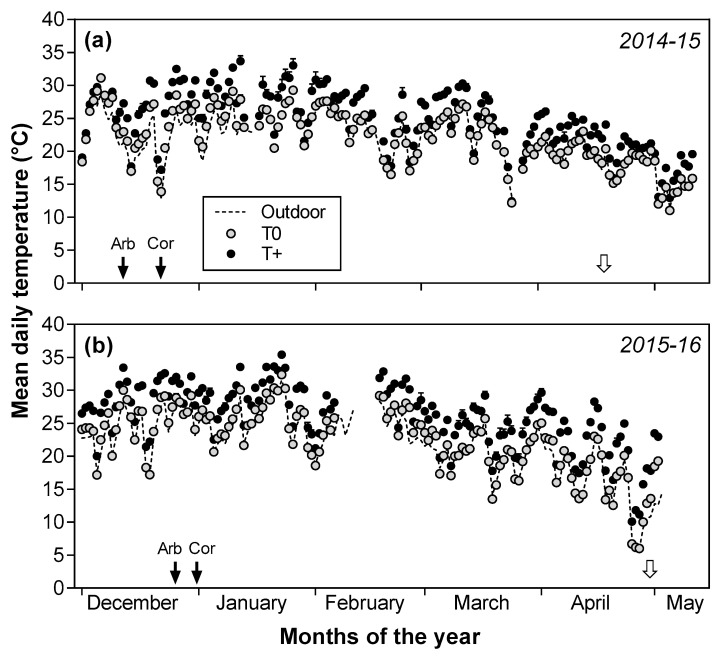
Mean daily air temperature in the control (T0) and warmed (T+) open top chambers (OTCs) and the ambient outdoor temperature during the oil accumulation phase in 2014–2015 (**a**) and 2015–2016 (**b**). Mean values ± SE (*n* = 8) of the temperature treatments are shown. Black arrows indicate the pit hardening dates for each cultivar. Empty arrows indicate the final fruit harvest dates. OTC temperatures were not recorded between 7–17 February and 1–3 May in 2015–2016 due to errors with the data logger, but both heating and ventilation systems were functioning.

The OTCs were located in an open field, and mean daily air temperature in the T0 and T+ OTCs fluctuated with ambient outdoor temperature during the warming period in both seasons (Figure 1). The control T0 temperature was less than 1 °C above the ambient outdoor temperature with mean daily temperature being 22.2 °C in T0 and 24.9 °C in T+ in 2014–2015 for a difference of 2.7 °C between T0 and T+ OTCs (Figure 1a). The mean temperature was similar in T0 in 2015–2016 with an average value of 22.5 °C, although the temperature was somewhat greater in T+ (26.1 °C) than in 2014–2015. Thus, the difference between the T0 and T+ OTCs was 3.6 °C in 2015–2016 (Figure 1b).

Similar to that reported in [20], other environmental variables of biological interest were measured in the OTCs. The atmospheric CO_2_ concentration in the OTCs averaged 425 ppm with no differences between the T0 and T+ OTCs. The photosynthetically active radiation (PAR) in the OTCs averaged about 75% of the ambient, outdoor values due to some absorption by the plastic sidewalls of the OTCs. Relative humidity (%) was about 5% less in the T+ OTCs than in the controls, and vapor pressure deficit was estimated to increase moderately (+35%) in the T+ OTCs as would be expected with elevated temperature.

### 2.2. Seasonal Patterns of Fruit Growth and Oil Accumulation 

**Figure 2:**
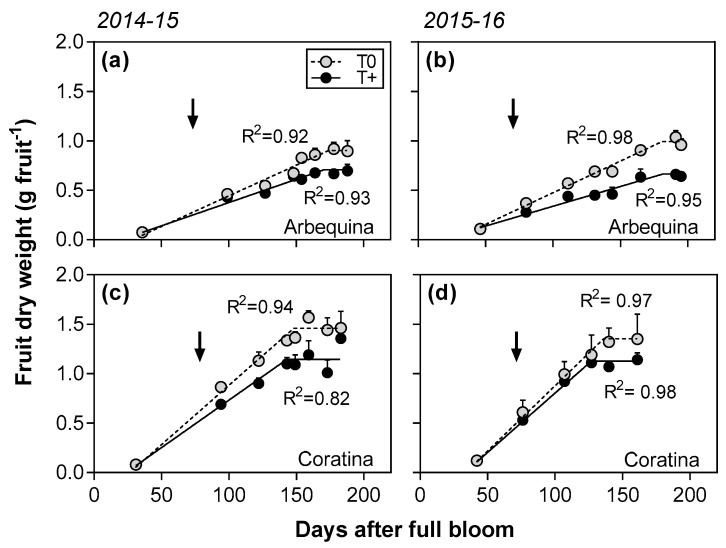
Fruit dry weight (g fruit^−1^) as a function of days after full bloom in control (T0) and warmed (T+) open top chambers for cvs. Arbequina and Coratina in 2014–2015 (**a**,**c**) and 2015–16 (**b**,**d**). The temperature treatment started on December 01 in both seasons (45-60 DAFB) and ended in early May after maximum oil concentration had been reached. The pit hardening date is indicated with black arrows. Mean values ± SE (*n* = 4) are shown.

In the two seasons evaluated, fruit dry weight versus days after full bloom (DAFB) was best fit to bilinear functions for each cultivar and temperature treatment combination (R^2^ = 0.82–0.98; Figure 2). In all cases, the pattern of fruit growth was characterized by a linear increase in dry weight followed by a plateau after reaching a constant, maximum weight value. The rate of the fruit dry weight increase was significantly reduced by about 20% in T+ trees compared to their respective T0 controls for both cultivars in 2014–2015 (*p* < 0.01; Table 1). In contrast, a significant cultivar x temperature interaction (*p* < 0.05) was apparent in 2015–2016 with the rate being 36% lower in cv. Arbequina T+ trees compared to T0 trees, while no difference was detected in cv. Coratina. Despite the rate differences, the duration of the fruit dry weight increase was similar between T+ and T0 for the two cultivars in 2014–2015. In 2015–2016, the duration was also not affected by the temperature treatment in cv. Arbequina, but it was 9 days shorter in the T+ than the T0 trees in cv. Coratina (interaction of *p* < 0.05). When comparing cultivars, cv. Arbequina fruit had lower growth rates and longer growth durations than cv. Coratina fruit (Table 1). The maximum fruit dry weight was 20 to 25% lower in T+ than in T0 trees in both seasons. As would be expected, the maximum fruit dry weight was always higher in cv. Coratina than cv. Arbequina. 

**Figure 3:**
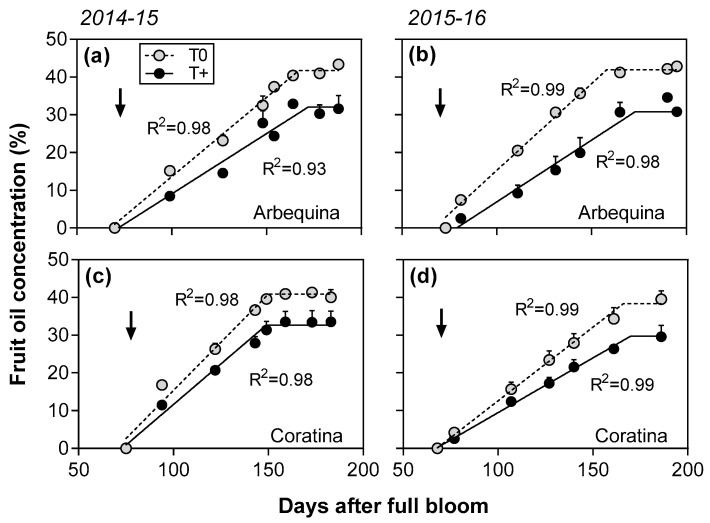
Fruit oil concentration (% dry weight) as a function of days after full bloom in control (T0) and warmed (T+) open top chambers for cvs. Arbequina and Coratina in 2014–2015 (**a**,**c**) and 2015–2016 (**b**,**d**). The temperature treatment started on 1 December in both seasons (1) and ended in early May after maximum oil concentration had been reached. The pit hardening date is indicated with black arrows. Mean values ± SE (*n* = 4) are shown.

Similar to fruit growth, oil concentration (i.e., % oil per fruit dry weight) versus DAFB was best fit to bilinear functions for each cultivar and temperature treatment combination in the two seasons evaluated (R*^2^* = 0.98–0.99; Figure 3). Fruit oil accumulation rate (i.e., oil concentration increase per day) was significantly reduced by 20% in T+ trees compared to T0 trees in both cultivars in 2014–2015 and 27% in 2015–2016 (*p* < 0.01; Table 1). In contrast, no significant differences due to the temperature treatment in oil accumulation duration were detected in either season. The oil accumulation duration was 20 days longer in cv. Arbequina than in cv. Coratina in 2014–2015, but no differences between cultivars were detected in 2015–2016. The maximum oil concentration was reduced about 20% in T+ trees compared to T0 trees in both cultivars and seasons. Lastly, cv. Arbequina had a higher oil concentration than cv. Coratina in 2015–2016 (*p* < 0.05).

**Table 1:** Best-fit bilinear regression parameters for fruit growth (g fruit-1) and oil accumulation (%) on a dry weight basis as a function of days after full bloom (DAFB) in control (T0) and warmed (T+) open top chambers for cvs. Arbequina and Coratina. Temperature treatments were applied from final fruit set to the end of the oil accumulation phase in 2014–2015 or 2015–2016. Values are means ± SE (*n* = 4). The statistical probability level for temperature treatment (temp), cultivar (cv), and their interaction (int) are given as not significant (*ns*), *p* < 0.05 (*), and *p* < 0.01 (**). Different italic letters are shown to separate means when significant differences (*p* < 0.05) for the cv x temp interaction were detected using Duncan’s test.

Season	Cultivar	Temp. Treatment	Fruit growth			Fruit Oil Accumulation		
			Rate ^a^ (mg day^−1^)	Duration ^b^ (DAFB)	Maximum Weight ^c^ (g)	Rate ^a^ (% day^−1^)	Duration ^b^ (DAFB)	Maximum Oil ^c^ (%)
2014–2015	Arbequina	T0	6.2 ± 0.3	174 ± 7	0.91 ± 0.06	0.42 ± 0.01	167 ± 3	42 ± 1
		T+	4.7 ± 0.3	171 ± 13	0.72 ± 0.07	0.32 ± 0.02	172 ± 6	32 ± 3
	Coratina	T0	12.0 ± 0.7	148 ± 10	1.47 ± 0.07	0.51 ± 0.02	150 ± 5	41 ± 1
		T+	9.6 ± 0.1	143 ± 6	1.16 ± 0.06	0.43 ± 0.01	149 ± 7	33 ± 3
		temp	**	*ns*	**	**	*ns*	**
		cv	**	*	**	**	**	*ns*
		int	*ns*	*ns*	*ns*	*ns*	*ns*	*ns*
2015–2016	Arbequina	T0	6.4 ± 0.3 *b*	181 ± 6 *a*	0.99 ± 0.06	0.46 ± 0.02	158 ± 4	42 ± 1
		T+	4.1 ± 0.6 *c*	182 ± 12 *a*	0.66 ± 0.04	0.33 ± 0.04	173 ± 9	33 ± 0
	Coratina	T0	13.2 ± 2.1 *a*	136 ± 9 *b*	1.36 ± 0.21	0.39 ± 0.03	166 ± 7	38 ± 2
		T+	12.0 ± 1.0 *a*	127 ± 6 *c*	1.13 ± 0.03	0.29 ± 0.02	170 ± 10	30 ± 2
		temp	*ns*	*ns*	**	**	*ns*	**
		cv	**	**	*	*	*ns*	*
		int	*	*	*ns*	*ns*	*ns*	*ns*

^a^ Rate is the bilinear regression slope (see Figure 2 and Figure 3); ^b^ Duration is days after full bloom when a constant, maximum value (plateau) was reached for the bilinear regression (see Figure 2 and Figure 3); ^c^ Maximum fruit weight and oil concentration are the constant final values at the end of the season (see Figure 2 and Figure 3).

### 2.3. Patterns of the Main Fatty Acids during Oil Accumulation 

**Figure 4:**
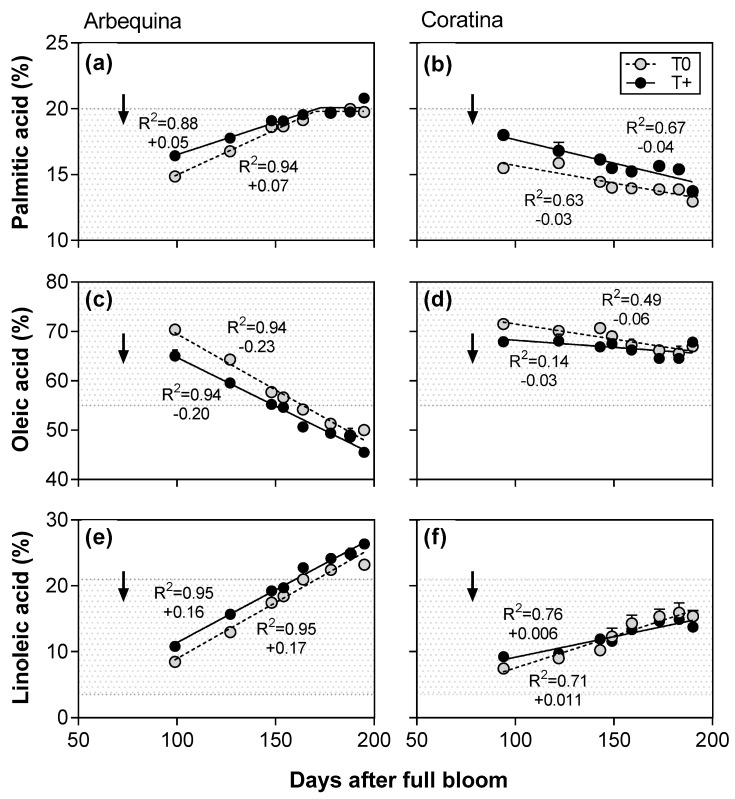
Main fatty acids (%) in fruit oil as a function of days after full bloom in control (T0) and warmed (T+) open top chambers for cvs. Arbequina (**a**,**c**,**e**) and Coratina (**b**,**d**,**f**) in 2014–2015. The temperature treatment started on 1 December in both seasons (45–60 DAFB) and ended in early May. The pit hardening date is indicated with black arrows for each cultivar and season. The gray background corresponds to values within the minimum and maximum limits established by the International Olive Council for extra virgin olive oil.

**Figure 5:**
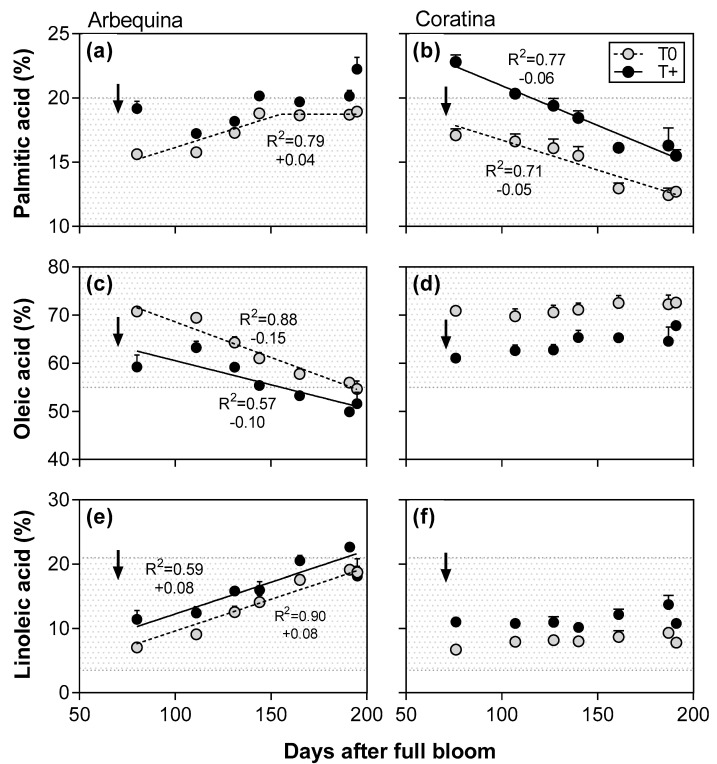
Main fatty acids (%) in fruit oil as a function of days after full bloom in control (T0) and warmed (T+) open top chambers for cvs. Arbequina (**a**,**c**,**e**) and Coratina (**b**,**d**,**f**) in 2015–2016. The temperature treatment started on 1 December in both seasons (45–60 DAFB) and ended in early May. The pit hardening date is indicated with black arrows for each cultivar and season. The gray background corresponds to values within the minimum and maximum limits established by the International Olive Council for extra virgin olive oil.

Depending on the fatty acid and cultivar, either linear or bilinear functions were best fit to the patterns of the main fatty acids for the two temperature treatments on a days after full bloom basis in both 2014–2015 and 2015–2016 (Figure 4 and Figure 5). In 2014–2015, palmitic acid showed a bilinear response in both T+ and T0 in cv. Arbequina with it increasing linearly until reaching a plateau at about 170 DAFB (Figure 4a). In contrast, palmitic acid in cv. Coratina decreased linearly during the entire experimental period at both temperature levels (Figure 4b). Oleic acid had the highest fatty acid percentages of all FAs, although it decreased linearly in both temperature treatments and cultivars (Figure 4c,d). The rate of decrease was slightly lower in T+ than T0 in each cultivar. Between cultivars, the cv. Arbequina showed a much greater rate of decrease than was observed in cv. Coratina during the experimental period. Linoleic acid increased linearly in both temperature treatments and cultivars, but the rate was more pronounced in cv. Arbequina (average of +0.17% day^−1^) than in cv. Coratina (+0.08% day^−1^) (Figure 4e,f). 

In 2015–2016, similar patterns were found for most of the fatty acids (Figure 5). However, palmitic acid in cv. Arbequina T+ did not fit to either a lineal or bilinear model (Figure 5a), and no relationships between DAFB and oleic or linoleic acids were found in cv. Coratina (Figure 5d,f).

### 2.4. Fruit Characteristics and Fatty Acid Composition at Harvest

Pulp and pit weight as well as pulp/pit ratio were determined to gain insight into how they might affect whole fruit oil concentrations (%). Pulp dry weight in T+ fruit was strongly reduced in both cultivars in 2015–2016 (*p* < 0.01; Table 2). In contrast, pit weight was only reduced slightly by the temperature treatment in cv. Coratina. Principally due to the reduction in pulp weight, the pulp/pit ratio was reduced by 43% in cv. Arbequina T+ fruit compared to T0, while no significant difference was detected in cv. Coratina (i.e., a cultivar x temperature interaction; *p* < 0.01). Fruit oil content, an indicator of both fruit growth and oil accumulation expressed as grams of oil per individual fruit, was much lower in T+ than in T0 at harvest in 2015–2016 (Table 2) and in 2014–2015 (−33% in cv. Arbequina; −26% in cv. Coratina). The fruit moisture (%) as well as the maturity index were not significantly affected by the temperature treatment when determined at harvest in 2015–2016 (Table 2).

At the end of the fruit oil accumulation period, the oleic acid percentage varied between 45–75% with lower values occurring in cv. Arbequina than in cv. Coratina (Table 3). In 2014–2015, warming caused a reduction of 4.5% points in oleic acid in cv. Arbequina T+ compared to its control T0, while in cv. Coratina no difference between temperature treatments was detected (cv. x temperature interaction; *p* < 0.01). In contrast, T+ trees of both cultivars showed a reduction in the oleic acid level compared to their respective T0 in 2015–2016. The second and third most abundant fatty acids were linoleic (9–26%) and palmitic (14–21%) acids, respectively, with both fatty acids being greater in cv. Arbequina than in cv. Coratina. Similar to oleic acid, linoleic acid showed a different response to warming depending on cultivar and season. Linoleic acid was 3.2% points higher in fruit oil of T+ than of T0 in cv. Arbequina in 2014–2015, but no significant difference between temperature treatments was detected in cv. Coratina (cv. x temperature interaction; *p* < 0.01). In 2015–2016, linoleic acid% was higher in T+ in both cultivars than in T0. The palmitic acid% was higher in the fruit oil of T+ trees than in their T0 controls in both cultivars and seasons, but the differences between temperature treatments were larger in 2015–2016.

## 3. Discussion

The expansion of olive production to new environments and global warming emphasize the need for controlled, experimental field studies to assess how fruit growth and oil accumulation respond to temperature in different olive cultivars. This information could be relevant to identify adaptation strategies that allow high olive oil production and quality under scenarios of crop expansion to warmer areas and global change. Under our climatic conditions in Argentina, daily mean air temperatures during the oil accumulation phase averaged about 25 °C when air temperature was increased by 2.7 to 3.6 °C in the T+ treatment of the open top chambers. A moderate increase in vapor pressure deficit (+35%) accompanied this temperature increase in the OTCs as has been previously reported [20] and would be expected due to global warming for our region [30]. Reductions in both fruit growth and oil accumulation rates under the T+ conditions ultimately led to large decreases in final fruit dry weight and oil concentration in cvs. Arbequina and Coratina. In contrast, the durations of fruit growth and the oil accumulation phase were little affected by experimental warming.

The reductions in fruit growth and oil accumulation rates under experimental warming are largely consistent with multiple environment and multiple season studies [4,11,15]. A previous study with six cultivars growing at three different altitudes in our region (NW Argentina) similarly found that both fruit growth and oil accumulation rates decreased when mean temperature increased [4]. In that study, oil concentration (%) decreased from 48.5 to 36.5% as mean daily temperature during oil accumulation increased from 23 to 27 °C. In a colder region (central Argentina), an evaluation of 10 cultivars during two growing seasons found that fruit oil concentration (%) was negatively associated with maximum daily temperature to some degree, but no significant correlations between fruit growth rates and weather variables were apparent [11]. Lastly, a comparison of potted, young olive trees of five olive cultivars positioned after fruit set at either a very warm or a moderate temperature location in Israel observed that most cultivars had lower fruit growth and oil accumulation rates at the warm desert location where daily maximum temperature often exceeded 40 °C during oil accumulation [15]. 

The durations of fruit growth and oil accumulation expressed as number of days are important because they determine the optimal timing of harvest in olive orchards. In central Argentina, fruit growth and oil accumulation duration were shorter with higher maximum daily temperatures during the oil accumulation period, and differences in duration between cultivars were also observed [11]. A multiple environment study with five cultivars in southern Spain found that high temperatures in the summer delayed the date at which maximum oil concentration was reached, while higher than normal temperatures in the autumn led to earlier dates of maximum oil concentration [13]. Additionally, cultivar did not affect the date of maximum oil concentration in that study. In warm northwestern Argentina, fruit growth and oil accumulation durations expressed on a thermal basis were similar between locations in two seasons, but durations in number of days were not determined [4]. In the current study, durations in number of days did not show differences between T+ and T0 trees in the OTCs with the exception of fruit growth duration in cv. Coratina in 2015–2016, which was 9 days shorter in the T+ trees. This shorter duration may have been more related to the unintentional selection of trees with somewhat less fruit number for the T+ treatment (250 ± 57) than for the near-ambient controls (422 ± 119). Although this difference was not statistically significant, low crop load has previously been shown to shorten fruit maturation period [31]. With respect to cultivar, the durations were consistently longer in cv. Arbequina than in cv. Coratina. Further research seems warranted to better understand the differences in response between regions. It is likely that the specific temperature regime over the course of the year and crop phenology of each region contribute to such differences. Furthermore, studies including a wider range of cultivars would be advantageous. 

Fruit oil content, which is defined as grams of oil per individual fruit, is useful in that it provides an integrated assessment of fruit growth and oil accumulation to experimental warming. Under our experimental conditions, the final fruit oil content was severely reduced by warming (3 °C) in both cultivars. Since warming began a few weeks before massive pit hardening when most of the pit growth had already occurred, the negative effects of elevated temperature on fruit oil content in both cultivars were largely related to decreases in pulp weight and its oil concentration. An estimation of pulp oil concentration (%) found that it was reduced by about −7.5% points in 2015–2016. Some experimental evidence with mesocarp tissue suggests that triacylglycerol synthesis is strongly reduced when temperature exceeds 30 °C [32], which appears to be related to gene repression [7]. The fruit oil content was also reduced by experimental warming to some degree due to an indirect “dilution effect” related to a significant reduction in the pulp/pit ratio in cv. Arbequina (−43%), but not in cv. Coratina (−17%). In other words, the greater relative weight of the lignified, woody pit with its low oil concentration (4.5%; [33]) compared to the pulp in T+ fruit further decreased fruit oil concentration in cv. Arbequina. Similar fruit oil effects due to changes in pulp/pit ratio were previously reported in response to crop load [31,34,35] or fertilization [36]. 

Low oleic acid, the main FA in the oil of olive fruit, is a common feature in warm NW Argentina compared to higher values in the Mediterranean Basin [3,4,29], and is a negative attribute for nutritional quality and oil shelf life. In the present study, the use of two temperature levels clearly showed that lower oleic acid in the T+ OTCs was especially evident early in the oil accumulation phase, and then the differences between the temperature treatments were either maintained or reduced towards harvest. Such initially large differences may have been related to accelerated fruit development or gene expression early in oil accumulation in the T+ fruit [7,37]. At final harvest, the response to experimental warming depended on the cultivar and season. In 2014–15, GxE interactions were observed for both oleic and linoleic acid contents with warming leading to a decrease in oleic acid (−4.5% points) and an increase in linoleic acid (+3.2% points) in cv. Arbequina, while cv. Coratina had similar levels of both FAs in the T+ and T0 control. In contrast, no GxE interactions were found in 2015–2016 with oleic acid decreasing and linoleic acid increasing under T+ in both cultivars. While oleic acid content in cv. Arbequina has previously been shown to be sensitive to temperature along latitudinal gradients in western Argentina [29,38], oleic acid in cv. Coratina was found to be relatively stable at different latitudes [38]. In the current study, the cv. Coratina only had lower oleic acid when the difference between the T+ and control temperatures in the OTCs was greater the second season (2.7 °C in 2014–2015; 3.6 °C in 2015–2016). Lower oleic acid was also observed in cv. Coratina in Israel at a high temperature site with the average temperature at that site being about 7 °C higher than that of a moderate temperature site [15]. Taken as a whole, the results suggest that oleic acid response to temperature is cultivar-dependent, and that large differences in temperature between locations or due to global warming are needed for changes in oleic acid to be observed in some cultivars.

## 4. Materials and Methods

### 4.1. Plant Material and Growing Conditions

A warming experiment was conducted from final fruit set until the end of the oil accumulation phase (December to early May) during the 2014–2015 and 2015–2016 growing seasons at the CRILAR-CONICET Experimental Field Station in La Rioja, Argentina (28° 48′ S, 66° 56′ W; 1325 m above sea level). A year prior to the first experimental season, self-rooted olive trees (cvs. Arbequina, Coratina) from a local commercial nursery (San Gabriel S.A.; La Rioja, Argentina) were transplanted to 30-l plastic pots containing a 5:2 sandy soil: perlite substrate at the field station. Irrigation was applied daily using drip irrigation to satisfy the tree water requirements plus soil evaporation. Fertilization with macro- and micro-nutrients was supplied monthly and weekly, respectively.

The trees were two-years old during the first experimental season in 2014–2015, and separate, untreated three-year old trees were employed in 2015–2016. Full bloom was determined in the springs of 2014 and 2015, and defined as the date when at least 50% of flowers were open (phenological stage BBCH 65) according to [39]. Full bloom occurred in 2014–2015 well before elevated temperature was applied on Oct. 1 in cv. Arbequina and Oct. 6 in cv. Coratina, while it was somewhat later in 2015–2016 (18 October in cv. Arbequina; 22 October in cv. Coratina). Massive pit hardening (PH) was measured as the date when the endocarp could no longer be transversely cut with a knife. It occurred in December two to four weeks after the experimental warming began. PH was 73 and 78 days after full bloom (DAFB) during 2014–2015 in cvs. Arbequina and Coratina; respectively, and 70 (cv. Arbequina) and 71 (cv. Coratina) DAFB during 2015–2016. Fruit number per tree in 2014–2015 was approximately 340 in cv. Arbequina and 200 in cv. Coratina. With older trees in 2015–2016, fruit number was about 610 in cv. Arbequina and 340 in cv. Coratina.

### 4.2. Temperature Treatments and Experimental Design

Two temperature treatments were established in the field using open top chambers (OTCs): a control (T0) that was less than 1 °C above the ambient outdoor temperature and an elevated temperature treatment (T+) with a target temperature of 4 °C above T0 [20]. The temperature treatments started on 1 December during both 2014–2015 and 2015–2016 and ended in early May after maximum oil concentration had been reached. The chambers were cuboid in shape (2 m × 1.5 m × 1.5 m) with the sidewalls being covered by polyethylene (150 μm thick; Premium Thermal Agrotileno PLDT221510, AgroRedes, Buenos Aires, Argentina). The T0 OTCs had a ventilation system to reduce passive heating originating from the polyethylene sidewalls. On sunny days, each T+ OTC was warmed using an external, 6-m-long plastic sleeve containing black painted stones through which warmed air was drawn into the OTCs by fans. In addition, an electric space heater activated electronically through a controller system (Cavadevices, Buenos Aires, Argentina) to provide heat during the night and on cloudy days. 

Air temperature was measured every 15 min by a temperature sensor (TC1047A, Microchip Inc., Chandler, AZ, USA) located at a height of 1.0 m (i.e, tree crown height) in the center of each OTC and recorded using a datalogger. As previously reported, other environmental variables including atmospheric CO_2_ concentration, photosynthetically active radiation (PAR), and relative humidity were measured periodically in the OTCs [20]. The CO_2_ concentration was measured at 1.0 m height in both the T0 and T+ OTCs from 9 to 15 h solar time on numerous days per season using two non-dispersive, infrared CO_2_ sensors (Cavadevices, Buenos Aires, Argentina). At the end of the 2014–2015 season, the PAR was measured over the course of the day in three empty OTCs at five different horizontal positions and at a height of 30 cm above the soil surface using a 1-m long, integrating light bar (BAR-RAD 100, Cavadevices, Buenos Aires, Argentina). Relative humidity (RH; %) was recorded at 30 min intervals on some days using humidity sensors connected to dataloggers (Cavadevices, Buenos Aires, Argentina). From both temperature and RH data, the absolute water vapor content and subsequently the vapor pressure deficit (kPa) were estimated. More details as well as photographs of the structure and operation of the OTCs can be found in [20]. 

The experimental design was a completely randomized block design with each of the four blocks containing four OTCs (i.e., a total of 16 OTCs). Within a block, each OTC contained one of four different treatment combinations (2 cultivars × 2 temperature levels). Four potted trees could be positioned in an OTC in individual soil cavities to reduce soil heating. Three trees per OTC were used the first season for the aims of this study, while two trees per OTC were used the second season when the trees were of greater size and fruit number.

### 4.3. Seasonal Patterns of Fruit Growth, Oil Accumulation, and Fatty Acid Composition

Fruit weight, fruit oil concentration (%) on a dry weight basis, and the main fatty acids in the oil were determined from successive fruit samples. In most cases, eight samples were taken during the season with the first sampling occurring a few days before the start of the experiment and the samples increasing in frequency towards the anticipated date of maximum oil concentration near the end of the season. Fatty acid composition was not determined for the first sampling date in both seasons. The samples were comprised of 20 fruit per OTC in cv. Arbequina and 10 fruit per OTC in cv. Coratina due to differences in fruit size between cultivars. The fruit samples were collected from two trees per OTC in 2014–2015 when the trees were small and one tree per OTC in 2015–2016. On average, about 30% of the fruit were removed per tree over the course of each season.

Each fruit sample per OTC was dried in a forced-air oven at 70 °C until constant weight was reached, and the average dry weight per fruit was determined (g fruit^−1^). Subsequently, the dried fruit from each sample were ground with an electric mill and the resulting 20–25 g of paste was used for determining the fruit oil concentration (%) on a dry weight basis by nuclear magnetic resonance (NMR, SKL-200 model, Spinlock, Córdoba, Argentina). As suggested by [40], the NMR instrument had been previously calibrated by comparing fruit oil concentrations measured by NMR with concentrations obtained by oil extraction using n-hexane with a Soxhlet apparatus. 

The fatty acid profile was determined by gas chromatography (PerkinElmer Clarus 500, Waltham, MA, USA) after extracting the oil from the dry paste of each sample at room temperature using n-hexane. An aliquot of oil was cold methylated in a basic medium (IOOC, 2013) and injected into the gas chromatograph fitted with a column (Zebron ZB-WAX, Phenomenex, Torrance, CA, USA) of 30 m length, 0.25 mm ID and 0.25 µm of film thickness. The carrier gas was hydrogen, and the injection and detector temperatures were 240 °C and 300 °C, respectively. The fatty acid proportions were determined by comparison with the retention times of known standards (AOCS-1, Sigma–Aldrich, St. Louis, MO, USA) and expressed as a percentage of the total amount of fatty acids.

### 4.4. Fruit Characteristics and Fatty Acid Composition at Harvest

All fruit were harvested from one tree per OTC once fruit oil accumulation had finished at the end of the season. These trees had not been used for the successive fruit samples during the course of the season. Maturity index (MI) was determined visually for 100 fruit from each harvested tree based on skin and pulp color [41]. These same samples were then oven-dried at 70 °C until constant weight, and the average fruit dry weight (g), fruit oil concentration (% dry wt.), and the individual fatty acids (%) were estimated using the methodology previously described for the successive fruit samples. The fruit oil content (g oil fruit^−1^) was also calculated as individual fruit dry weight multiplied by oil concentration. 

The pulp/pit ratio was determined from a separate sample of 30 fruit per tree at the end of the 2015–2016 season. These samples were oven-dried at 70 °C and fruit dry weight was determined with a precision balance. The fruit were then heated in a water bath until it was possible to physically separate the pulp from the pit. The pits were subsequently re-dried and re-weighed. Pulp dry weight per fruit was obtained as the difference between the fruit dry weight and that of the pit for each sample, and the pulp/pit ratio was calculated. Finally, the pulp oil concentration (%) on a dry weight basis was estimated from the pulp/pit ratio, the fruit oil concentration (%), and assuming that the pit (endocarp + seed) represented 4.5% of the total fruit oil [33]. Although the pit oil (%) may decrease with increasing temperature [42], any resulting error in estimating the pulp oil (%) would be small due to the low percentage of the total oil from the pit. 

### 4.5. Statistical Analyses

Best-fit relationships for fruit weight, fruit oil concentration, and the main fatty acids as a function of DAFB were determined for the successive fruit samples of each cultivar (Prism version 6.01, GraphPad Inc., San Diego, CA, USA). The potential relationships evaluated were bilinear, sigmoid, or linear with each data point representing the average of the four OTCs per temperature level. When considering bilinear relationships, rate was defined as the linear slope before a constant maximum plateau was reached, and duration was the number of DAFB to reach the plateau. Fruit growth and oil accumulation rates, durations, and the final maximum oil concentration values obtained from the best-fit relationships as well as final harvest data were analyzed using two-way ANOVA (InfoStat Versión 2018, Universidad Nacional de Córdoba, Argentina). Duncan’s post-test was employed to separate means when the cultivar x temperature treatment interaction was significant (*p* < 0.05).

## 5. Conclusions

Whole olive trees were warmed in the field to evaluate fruit growth, oil accumulation, and main fatty acid synthesis. The use of control and warmed OTCs in the field made it possible to more directly assess the effects of air temperature in comparison to multi-site studies that cannot separate completely the responses to air temperature from those of other environmental factors. A daily increase of about 3 °C led to significant reductions in both fruit growth and oil accumulation rates in two of the most common cultivars worldwide (cvs. Arbequina, Coratina). Consequently, final fruit weights and oil concentration (%) were reduced at harvest with warming as was fruit oil content (g oil per individual fruit). In contrast, the duration (days) of fruit growth and oil accumulation was most often not affected under our warm, climatic conditions. Such information could be of interest for simulation modeling related to global warming. Further research is recommended concerning the duration of the oil accumulation phase given that duration has been shown to be affected by environment in some other regions. Experimental warming also diminished oil quality by reducing oleic acid content in a cultivar and season-dependent manner. Studying how fruit growth and oil accumulation respond to adaptation strategies against increasing air temperatures should be a priority in both young and mature olive trees of numerous cultivars given crop expansion to new regions and future climate scenarios. Modifications in orchard design including row orientation in new orchards and foliar particle film application (e.g., kaolin) to increase canopy reflectance in existing orchards may reduce canopy temperatures and improve yields.

## Figures and Tables

**Table 2:** Fruit characteristics at harvest in control (T0) and warmed (T+) open top chambers for cvs. Arbequina and Coratina in 2015–2016. Temperature treatments were applied from final fruit set to the end of the oil accumulation phase. Values are means ± SE (*n* = 4). The statistical probability level for temperature treatment (temp), cultivar (cv), and their interaction (int) are given as not significant (*ns*), and *p* < 0.01 (**). Different italic letters are shown to separate means when significant differences (*p* < 0.05) for the cv x temp interaction were detected using Duncan’s test.

Cultivar	Temp. Treatment	Fruit Dry Weight (g)			Fruit Oil Content (g fruit^−1^)	Fruit Moisture (%)	Maturity Index (0–7)
		Pulp	Pit	Pulp/Pit			
Arbequina	T0	0.71 ± 0.06	0.28 ± 0.01 *c*	2.48 ± 0.15 *a*	0.41 ± 0.03	58.5 ± 0.6	3.8 ± 0.6
	T+	0.37 ± 0.03	0.26 ± 0.01 *c*	1.41 ± 0.10 *c*	0.20 ± 0.02	61.7 ± 2.4	3.3 ± 0.3
Coratina	T0	1.20 ± 0.12	0.57 ± 0.03 *a*	2.09 ± 0.12 *b*	0.77 ± 0.08	50.2 ± 2.0	0.5 ± 0.1
	T+	0.81 ± 0.04	0.47 ± 0.03 *b*	1.73 ± 0.03 *bc*	0.44 ± 0.02	55.9 ± 2.3	1.0 ± 0.3
	temp	**	**	**	**	*ns*	*ns*
	cv	**	**	**	**	**	**
	int	*ns*	**	**	*ns*	*ns*	*ns*

**Table 3:** Major fatty acids percentages in fruit oil at harvest in control (T0) and warmed (T+) open top chambers for cvs. Arbequina and Coratina. Temperature treatments were applied from final fruit set to the end of the oil accumulation phase in 2014–2015 and 2015–2016. Values are means ± SE (*n* = 4). The statistical probability level for temperature treatment (temp), cultivar (cv), and their interaction (int) are given as not significant (*ns*), *p* < 0.05 (*), and *p* < 0.01 (**). Different italic letters are shown to separate means when significant differences (*p* < 0.05) for the cv x temp interaction were detected using Duncan’s test.

Season	Cultivar	Temp. Treatment	Major Fatty Acids (%)		
			Palmitic	Oleic	Linoleic
2014–2015	Arbequina	T0	19.8 ± 0.1	50.0 ± 0.8 *b*	23.2 ± 0.5 *b*
		T+	20.8 ± 0.2	45.5 ± 0.9 *c*	26.4 ± 0.5 *a*
	Coratina	T0	12.9 ± 0.4	67.0 ± 1.1 *a*	15.4 ± 0.9 *c*
		T+	13.7 ± 0.2	67.8 ± 0.6 *a*	13.7 ± 0.4 *c*
		temp	**	*	**
		cv	**	**	**
		int	*ns*	**	**
2015–2016	Arbequina	T0	19.5 ± 0.7	54.7 ± 1.6	19.1 ± 0.5
		T+	22.3 ± 0.9	51.6 ± 2.4	22.7 ± 0.7
	Coratina	T0	12.7 ± 0.3	74.7 ± 0.8	9.3 ± 0.8
		T+	15.5 ± 0.5	68.5 ± 0.4	13.8 ± 1.4
		temp	**	*	**
		cv	**	**	**
		int	*ns*	*ns*	*ns*

## Data Availability

Data available from authors on request.
