# Correction: Miserere et al. Influence of Experimental Warming on the Rate and Duration of Fruit Growth and Oil Accumulation in Young Olive Trees (cvs. Arbequina, Coratina). *Plants* 2023, *12*, 1942

**DOI:** 10.3390/plants15030393

**Published:** 2026-01-28

**Authors:** Andrea Miserere, Peter S. Searles, M. Cecilia Rousseaux

**Affiliations:** 1 Centro Regional de Investigaciones Científicas y Transferencia Tecnológica de La Rioja (CRILAR-Provincia de La Rioja-UNLaR-SEGEMAR-UNCa-CONICET), Entre Ríos y Mendoza s/n, Anillaco 5301, La Rioja, Argentina; amiserere@conicet.gov.ar (A.M.); psearles@conicet.gov.ar (P.S.S.); 2 Instituto de Investigación y Desarrollo Agropecuario (IIDA), Departamento de Ciencias y Tecnologías Aplicadas (DACTAPAyU), Universidad Nacional de La Rioja (UNLaR), Av. Luis M. de la Fuente s/n, Ciudad Universitaria de la Ciencia y de la Técnica, La Rioja 5300, La Rioja, Argentina; 3 Departamento de Ciencias Exactas, Físicas y Naturales (DACEFyN), Universidad Nacional de La Rioja (UNLaR), Av. Luis M. de la Fuente s/n, Ciudad Universitaria de la Ciencia y de la Técnica, La Rioja 5300, La Rioja, Argentina

In the original publication [1], there was a missing reference by Breton et al. The authors and the Academic Editor believe that citing this reference makes the literature review in the introduction section more complete and provides a fuller context for the authors’ study. The reference has now been added as reference [16], and the subsequent reference numbers have also been updated. The authors state that the scientific conclusions are unaffected. This correction was approved by the Academic Editor.

16. Breton, C.; Souyris, I.; Villemur, P.; Berville, A. Oil Accumulation Kinetic along Ripening in Four Olive Cultivars Varying for Fruit Size. *OCL* **2009**, *16*, 58–64. https://doi.org/10.1684/ocl.2009.0236.
